# Alveolar Abscess

**Published:** 1868-07

**Authors:** W. H. Shadoan


### ALVEOLAR ABSCESS.

BY DR. W. H. SHADOAN.

ALVEOLI.—Semi-circular canals or grooves into which the teeth are set. Their size and shape are determined by the teeth that occupy them.

ABSCESS.—From Abscedo, I depart or separate from, loss of substance, a gathering or rising, a collection of pus in a cavity, the result of a morbid process or action in the parts. The French have various distinctive terms for abscess, as *Ab'ces chaudaign sondain*, is one which follows violent inflammation; *Ab'ces froid chronique scrofulux*, cold, chronic or scrofulous abscess, one which is the result of chronic or scrofulous inflammation; *Ab'ces par congestione*, diasthese-que, symptomatic abscess, one which occurs in a part, at a distance from the inflammation by which it is occasioned. "Some writers are of opinion that pus is formed by the arterial system, and is deposited by way of excretion in the inflamed parts; others, that it is formed by the destruction of the solid parts." It seems to be a degeneration of the liquor sanguinis and exudation corpuscles. "In Alveolar Abscess we have the whole range of structure involved, from unpronounced amorphous, mucus mass, or chaotic materials in the juices of the flesh, from which arise and by which are nourished the neural and muscular fibrillæ, the vascular and osseous, no less than the glandular and dermal tissue. If then all these must be involved in destruction, just in the ratio of the size of the sac, in every case of matured alveolar abscess, is it not of some moment to us to be able to detect the order of its inception and progress from its first beginning to its most unmistakable presence? Where then is the point of departure from normal activity? Is it in the juices of the flesh? Or is it in the granular living contents of the cells? Or may it not have its inception, in a refusal on the part of the formed material of the cell wall, to afford free transit into and out of the parenchyma of the cell to the pubulum, or juices upon which it subsists? Although it may be clear to him who has investigated this subject, that all departures from healthy action take their origin in the neural sea, or juices of the flesh; yet it is difficult to prove this to the uninitiated mind short of laborious and tedious detail of untrient activities. The oneness of this sea throughout the whole range of the body within the outer pellicle or skin, whether that body be large or small, composed of one organ or many divisions, their sustenance from this elemental mass, renders the whole body or any part of it, subject to change in accordance with the extent of the application and the force of the disturbing agent." *

There is very little difference between alveolar abscess and abscesses in general; the former has its origin within the alveolar border, while the latter may have their origin on the outer surface of the osseous system and in the soft parts.

Abscess is the most common affection to which the alveolus is subject. "Its effects are always exceedingly pernicious," † not only to the sockets of the teeth thus affected, but to the gums, and very often the health is largely affected thereby. When severe inflammation of the lining membrane

---

* Prof. Atkinson on Alveolar Abscess.

† Harris' Principles and Practice of Dental Science.

of the tooth or the alveolus is produced, causing the death of one or more cells which suppurate and form a pocket, there is an effusion of coagulable lymph, which will harden and form a sac, which attaches itself to the tooth or alveolus at the point of inflammation. This may be averted even after the sac is partially formed, by interrupting or cutting off the poisonous food or supply which feeds the disease. The cells forming the walls of the nucleated abscess having the least power of resistence, give way first, and determine the direction of the abscess. Cold abscess may readily be detected by its appearance, which is recognized by the parts being enlarged, of a soft or spongy appearance, with very little tenderness and slight constitutional disturbance. It progresses slowly, and is found in persons of low vitality and of a scrofulous temperament. The warm variety is almost the exact opposite of the cold, the parts swell, are red, and very tender to the touch. There is a great variety ranging from cold to warm; some will be intermediate, while others will approximate the one or the other extreme.

In evacuations of the cold or chronic variety the pus will be found thick and poorly defined, with very little traces of blood, laying close to the surface, showing a slow development and little variety. It is not so with the warm, the pus is more fluid, with traces of blood, and on evacuation will be found coming from the middle, while red blood will be discharged from the surface cut or walls of the tumor. The last occur in persons of sound constitution, are rapid, and of easy cure, if not complicated with other diseases, while the cold is, as before stated, slow and stubborn, requiring skillful treatment, or in other words, very tedious of cure, often requiring the best skill, and in some cases, resisting curative effort altogether.

### DIAGNOSIS.

The correctness of the diagnosis will depend somewhat upon three things, (viz:) Acuteness of perception, the stage of the affection, and the characteristics of the patient. In its earlier stages, in some peculiar constitutions, diagnosis is very difficult. But to the close observer and the thoroughly conversant it is not so, especially with the acute variety. The presence of abscess may, by some, be detected while yet in its primative or cell state, and in such a case cure is almost certain. The following may be considered some of the unmistakable signs of abscess: In the earlier stages redness of the gums, extreme tenderness of the tooth to the touch, swelling of the gums, &c. At a later period the symptoms become more prominent. Elongation and loosening of the tooth, an increased size of the gum over the point affected, a great rush (apparently) of blood to the part on taking a recumbent position; increase of pain in the parts, a fistulous opening through the gums, cheek, jaw, or at any other point where the pus may be conveyed by the aid of a suture or other channel, susceptible of being traversed by pus. These are the most prominent signs of alveolar abscess, and may be regarded as reliable. The cause and duration of the disease will depend upon the constitutional health of the patient, of the susceptibility to abscess, the stage of the disease when the treatment is commenced, and the kind of treatment adopted.

The warm variety is the most rapid in its course, and the cold is the slowest; the intermediate, in temperature, will vary between the two classes above named in duration. The general health always strongly influencing either variety or class of abscess.

CAUSES.

The causes of alveolar abscess are very numerous. A lack of power, on the part of the system, to take up and appropriate the nutritious food contained in the juices. The presence of irritating matter, dead nerve membrane, dead roots of teeth, mechanical violence, sudden and repeated transitions of temperature, and any other cause that produce acute inflammation. Irritating matter may be secreted at the point of the root of a tooth in which the nerve is dead, and as long as the canal of the root or roots is kept open there may be no injurious effects, but as soon as the avenues of escape are closed, by any means whatever, the escape of the matter is checked, and as the secretion is still kept up it will soon form, in such quantities, as to produce pressure on the lining membrane of the socket, and a high state of inflammation and congestion is set up. It is not always the case that a dead tooth has a decaying nerve, in some cases the nerve is entirely gone, yet a secretion may exist at the apicial foramin, which, if not allowed to escape, will produce the same effect that a dead pulp will. It is one of the most difficult points in pathology to ascertain just how much inflammation will be tolerated. If the recuperative powers be equal to the inflammatory conditions, one will poise the other, and as one predominates so will be the result.

In some cases there will be evil resulting from the slightest disease, while at other times it will take almost death itself in the parts to produce any change whatever. This is often manifested in other things; sometimes men die from slight scratches, and at other times they may be torn almost to pieces and yet live. I remember, during the late rebellion having seen a man who had received thirteen wounds, either of which seemed sufficient to have killed him; yet he wholly recovered. I allude to this only to show how much the system is capable of resisting at some times as compared with other times. We are all aware that the system can not endure as much at one time as it can at another. This depends somewhat upon the condition of the mind. An abscess is often produced by the simple operation of filling a tooth at an improper time, for instance, when the system is in a reduced condition, or when there is inflammation in the parts or any other unhealthy condition, that contributes to the formation of alveolar abscess. To attempt a clear and concise description of all the circumstances that tend to produce or contribute to the formation of abscess would be a tedious as well as unprofitable undertaking; the above refers mainly to roots of teeth and teeth in which there is no nerve membrane. The next class of causes that claim our attention are those in which the nerve membrane is yet remaining. A tooth may be slightly decayed, enough so to expose the pulp, and the irritation thereof from contact with the air, from chemical action or otherwise, may be sufficient to produce inflammation in the membrane, and this, if not relieved by topical applications, usually results in the death of the pulp. The power of recupuration in the pulp of a tooth is very low at best, and when inflamed from contact with the air and fluids of the mouth, death is almost sure to follow. The engorgement may be relieved by stimulants or by drawing the blood from this point to some other, thus permitting the part to recover. This may be done by the use of leeches, counter-irritants, activity of body or mind, or both, or any other act that will change the current of blood to some other point. It takes very little irritation in some persons, and especially at certain times to produce inflammation at the apex of the roots of teeth sufficient to cause an abscess, and the conditions mentioned above seem peculiarly to favor this result. There are other cases where the nerve of a tooth is not exposed, but the decay approaches very closely, the cavity is cleansed and filled, and the thermal changes in the filling, together with the close proximity of the same to the pulp, will cause irritation and inflammation; and as the tissue is encased in a firm and unyielding chamber the only egress is through the foramin of the root, and whether the cavity is filled or not an abscess may form. After a tooth is filled the nerve often dies from inflammation produced by the operation. Abscesses may be formed and exist for a long time without being apparent, and in persons of good health, have existed and been cured by nature alone. I have seen cases where there had been abscess and no external signs of lesion, and the persons themselves had no knowledge of its existence, clearly showing to my mind that an abscess may exist and be radically cured by nature alone. The next among the causes that we shall mention is mechanical violence; this, like many other causes, has its peculiarities. Mechanical violence may be in any direction that will bruise the periosteum of the tooth. But the most favorable is the lateral. You may force a tooth in the socket with such violence that it will bruise or otherwise wound the membrane, and cause inflammation. It may first be in the form of periostitis, but if the inflammation is not allayed a plasma will be wept out and matter formed; but as before stated the most favorable kind of violence for the production of abscess is the lateral. Strike a tooth on either side and not only bruise the investing membrane, but the nerve itself is liable to be injured, either of which will cause thickening of the membrane and produce the same result. An alveolar abscess is sometimes caused by simple inflammation of the periosteum, this is often the result of sudden transitions of temperature. These are some of the exciting causes, there are others that might be named, but for the present we will omit them. There are general or constitutional causes which contribute largely to the formation of abscess. Persons of a manifest inflammatory diathesis or those in which there is considerable local inflammation from some local exciting cause. Those of a manifest strumous diathesis and persons living in miasmatic districts are more likely to be attacked than those of a healthy condition.

TO BE CONTINUED.

